# A five years malaria surveillance data analysis of North Shewa zone, Amhara region, Ethiopia: July 2018 to June 2023

**DOI:** 10.1186/s12936-024-05006-w

**Published:** 2024-06-15

**Authors:** Tebabere Moltot, Girma Bekele, Zenebe Abebe Gebreegziabher, Tesfansh Lemma, Moges Sisay, Mulualem Silesh, Melkam Mulugeta, Legesse Demissie, Tirusew Nigussie Kebede, Birhan Tsegaw Taye

**Affiliations:** 1 School of Nursing and Midwifery, Asrat Woldeyes Health Science Campus, Debre Berhan University, Debre Birhan, Ethiopia; 2 School of Public Health, Asrat Woldeyes Health Science Campus, Debre University, Debre Birhan, Ethiopia; 3 School of Medicine, Asrat Woldeyes Health Science Campus, Debre Berhan University, Debre Birhan, Ethiopia

**Keywords:** Data analysis, Malaria surveillance, North Shewa zone, Amhara, Ethiopia

## Abstract

**Background:**

Malaria is a critical public health concern in Ethiopia, with significant socioeconomic consequences. Malaria data trend analysis is essential for understanding transmission patterns and adopting evidence-based malaria control measures. The purpose of this study was to determine the 5 year distribution of malaria in North Shewa zone, Amhara region, Ethiopia, in 2023.

**Methods:**

A descriptive cross-sectional study design was employed to analyse the 5 year trend of malaria surveillance data in the North Shewa zone of the Amhara regional, Ethiopia, spanning from July 2018 to June 2023. The malaria indicator data were gathered from the zone’s public health emergency management database. Malaria data from the previous 5 years was collected, compiled, processed, and analysed using Microsoft Excel 2019.

**Results:**

Among a total of 434,110 suspected cases 47,889 (11.03%) cases were confirmed as malaria, with an average annual malaria incidence rate of 4.4 per 1000 population in the Zone. Malaria cases exhibited an increase from Epidemiological Week (Epi week) 37 to Epi week 49 (September to November) and again from Epi week 22 to week 30 (May to July). Individuals aged 15 and above, and all districts in the Zone except Angolela were notably affected by malaria.

**Conclusion:**

Despite implementing various measures to reduce malaria incidence, the disease continues to persist in the zone. Therefore, the Zone Health Department should intensify its preventive and control efforts.

## Background

Globally, Malaria prevention and treatment programs have saved over 11 million lives since 2000, and malaria deaths in Africa have been decreased by 36% between 2000 and 2021 [1].

Despite this, malaria remains a major public health and life-threatening parasite illness [2]. In 2021, 3.2 billion people—almost half the world’s population—are at risk, 619,000 people died from malaria [1]. Globally there were an estimated 249million malaria cases in 2022, up from 5 million in 2021 [3]. The African continent is responsible for 95% of all malaria cases and 96% of all malaria deaths [4]. Furthermore, the survey revealed that 80% of all malaria deaths in the African region occurred among the next adult generation (children under the age of five) [5], and that malaria costs Africa nearly $12 billion per year [1].

Malaria cases spread more broadly during normal monsoon years than during drought years, owing to a more favourable environment for mosquito breeding [6, 7]. The unpredictable and seasonal transmission pattern of malaria is influenced by altitude and rainfall. In Ethiopia, the majority of regions have peak malaria transmission from September to December, following the primary wet season (June to August), and the second minor transmission occurs from April to June, following a brief rainy season (February to March) [8, 9]. It is estimated that 75% of Ethiopia’s landmass is malarial, and 68% of its population is at risk of getting the illness. The most severe form of malaria is caused by *Plasmodium falciparum*, responsible for 60–70% of malaria cases in the country [10].

Ethiopia has embarked on a malaria prevention and control programme with the ambitious goal of eliminating the disease by 2030 [11, 12]. Despite the implementation of various prevention and control strategies, malaria remains a significant public health challenge in the country [13].

Recent findings have indicated the emergence of a newly imported *Anopheles* species, *Anopheles stephensi,* along with increasing trends in malaria cases. Factors such as disruptions to malaria services during the COVID-19 pandemic in 2020, and ongoing conflicts in the country have contributed to the region’s malaria burden [14]. Malaria is closely monitored as an epidemic-prone disease through the Public Health Emergency Management (PHEM) surveillance system. Continuous data analysis is crucial for early outbreak detection, tracking trends, and evaluating control programme effectiveness [15]. The North Shewa zone is one of a high malaria endemic area within the region [16, 17]. Therefore, this study aims to assess the 5 year distribution of malaria cases in the zone: from July 2018 to June 2023.

## Methods

### Study setting, period and design

A descriptive cross-sectional study design was employed to analyse the 5 year trend (from July 2018 to June 2023) of malaria surveillance data in the North Shewa zone, located within the Amhara region. Ethiopia. The zone spans an area of 15,936.13 square kilometres with a population density of 115.3 people per square kilometre [18]. It encompasses all three broad agro-ecological zones: Kolla (hot lowlands below 1000 m altitude), Weyna-Dega (midlands between 1000- and 1500 m altitude), and Dega (cool temperate highlands 1500 m above sea level). Within the North Shewa zone, there are twenty-three districts, eleven hospitals, ninety-two health centres, and more than 380 health posts. The zone includes three general hospitals and one referral hospital, all of which directly report surveillance data to the North Shewa zone’s PHEM) directorate.

### Population

The source population for this study comprised individuals seeking healthcare services at facilities within the North Shewa zone between July 2018 and June 2023. The study population specifically included all reported cases of malaria diagnosed within health facilities in the North Shewa zone during the same time frame.

### Eligibility criteria

The study included confirmed and clinically treated cases of malaria over a 5 year period, while incomplete data were excluded from the analysis.

### Study variable

The dependent variable was number of malaria cases, whereas the independent variable were years, age, pregnancy status, woreda/hospitals, Epi-weeks.

### Operational definition

**Suspected case:** any person with fever or fever with headache, back pain, chills, rigor, sweating, muscle pain, nausea and vomiting were considered as suspected cases for malaria [17].

**Confirmed case:** suspected case confirmed by rapid diagnostic test (RDT) or microscopy for *Plasmodium* species [17].

### Data collection tools and procedure

First, written permission was obtained from the North Shewa zone’s health department. Subsequently, 5 years of secondary data spanning from July 2018 to June 2023 were acquired from the zone’s health department’s PHEM directorate. Following this, an Excel extraction sheet was prepared, and the required data were retrieved and compiled from the reporting database using pivot tables.

### Data quality control, processing and analysis

Descriptive data analysis was done by using Microsoft office excel-2019. Before analysis the data were checked for completeness and consistency. And also, during analysis a team of data analysts to review and validate data at different stages of the analysis process were done. The data were summarized and displayed by using table and different graphs.

## Results

### Malaria surveillance data analysis by time

#### Five-year malaria incidence in North Shewa zone

Out of a total of 434,110 probable fever cases examined through rapid diagnostic test (RDT), microscopy, and clinical assessment, 47,889 cases (11.03%) were confirmed as malaria. The proportion of malaria admissions (inpatient malaria cases) rose from 0.015% in 2018 to 0.35% in 2023. Throughout this reporting period, one death was recorded in Epi week 46 of 2020 in the Merhabete district, resulting in a case fatality rate of 0.002% in the zone. On average, 8978 malaria cases were reported annually to the PHEM, with numbers ranging from 6710 in 2019 to 12,790 in 2020. Over the same timeframe, the zone’s average annual malaria incidence rate stood at 4.4 cases per 1000 inhabitants (Table 1).

**Table 1:** Annual malaria incidence rate/1000 population per years in North Shewa Zone, Amhara, Ethiopia, July 2018–June 2023

Years	Total population	Malaria case	IPD*_malaria case	Incidence rate/1000 population/year	Case fatality rate
July 2018–June 2019	2056131	6710	1 (0.015%)	4	$\frac{1}{47889}*100=0.00$ 2
July 2019–June 2020	2283969	12799	22 (0.172%)	6	
July 2020–June 2021	2335205	12037	19 (0.16%)	5	
July 2021–June 2022	2370487	9301	21 (0.26%)	4	
July 2022–June 2023	2388122	7042	25 (0.35%)	3	

^*^IPD Inpatient department

#### Trends of malaria species

**Fig. 1:**
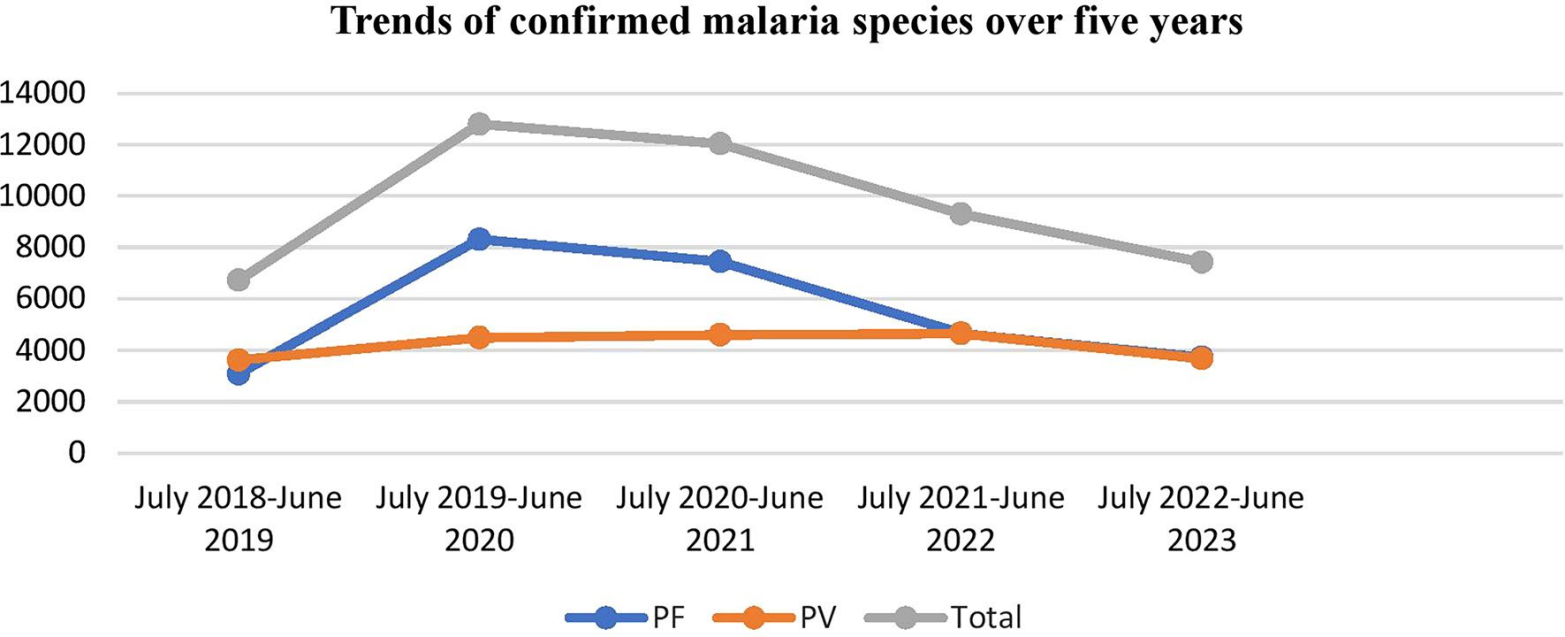
The five years trends of malaria by *Plasmodium* species in North Shewa zone, Ethiopia, July 2018–June 2023

According to PHEM data from the previous 5 years, from a total 47, 889 confirmed malaria cases, 27010 (56.4%) of confirmed malaria patients had *P. falciparum* and 43.6% had *Plasmodium vivax*. *Plasmodium falciparum* increased dramatically from July 2019 to June 2021, then decreased. Throughout a 5 year period, the trends in *P. vivax* malaria cases were nearly stable (Fig. 1).

#### Trends of malaria by epidemiologic weeks

**Fig. 2:**
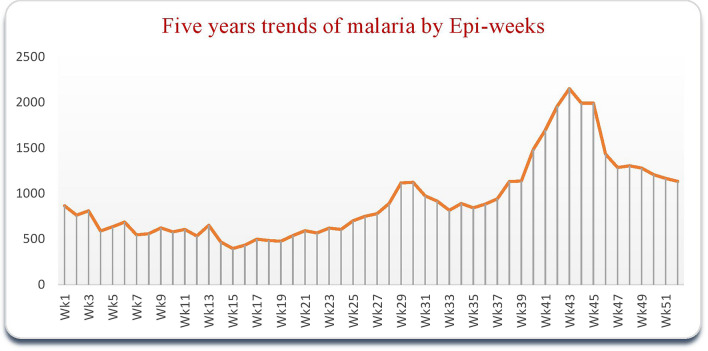
Trend of five year aggregated total malaria cases with similar WHO weeks of the past five years of North Shewa Zone/July 2018–June 2023

**Fig. 3:**
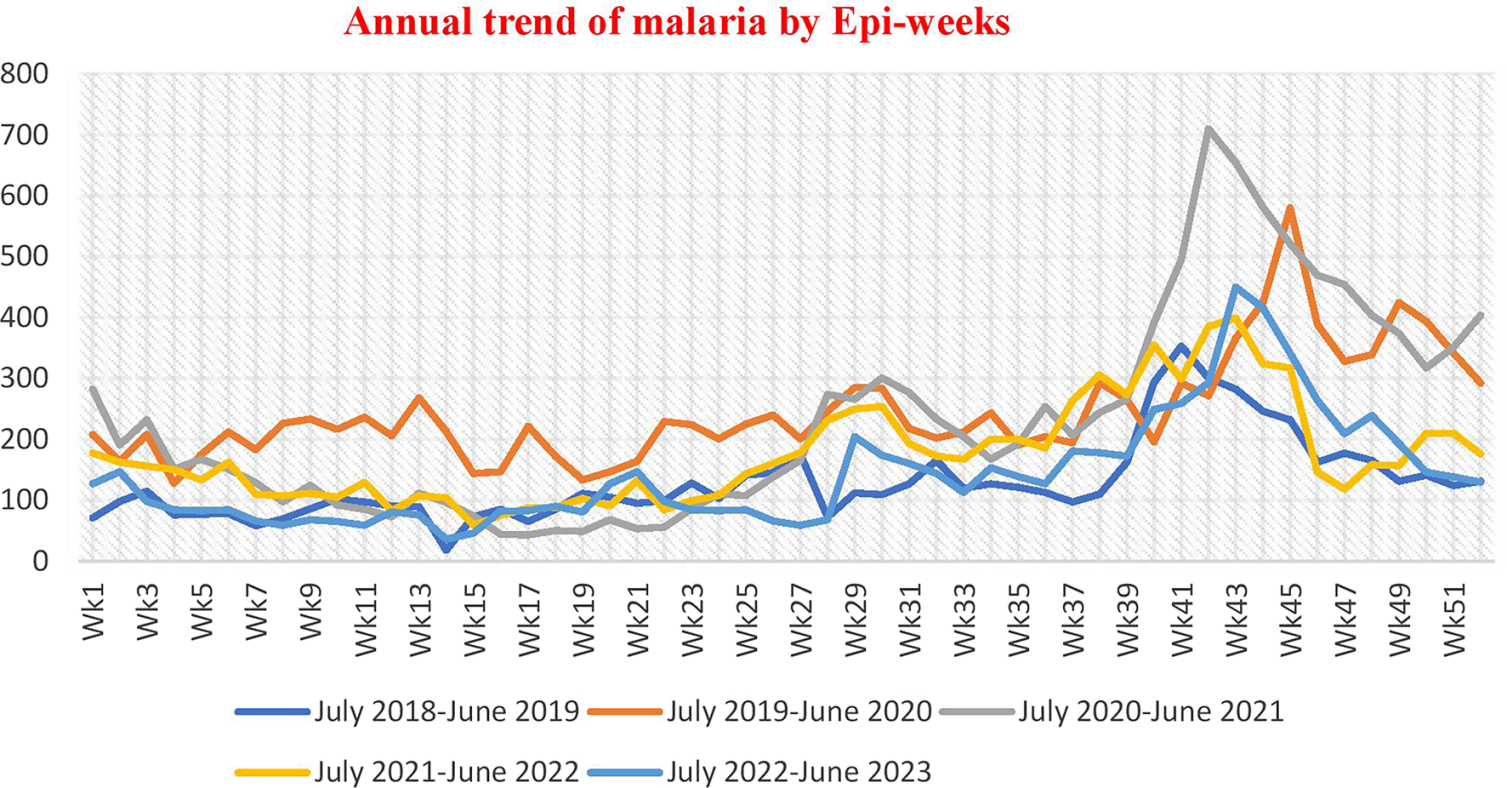
Each year of total malaria cases with WHO Epi-weeks of the past 5 years of North Shewa Zone/July 2018–June 2023

The systematically gathered and analysed malaria surveillance data confirmed that the trend of malaria cases began to grow from Epi week 37 to 49 (September to November). Then again from Epi week 23 to 29 (May to July) (Fig. 2). A higher/peak of incidence of malaria were observed from July 2020 to June 2021 (Fig. 3).

### Malaria surveillance data analysis by person

#### Analysis by age

**Fig. 4:**
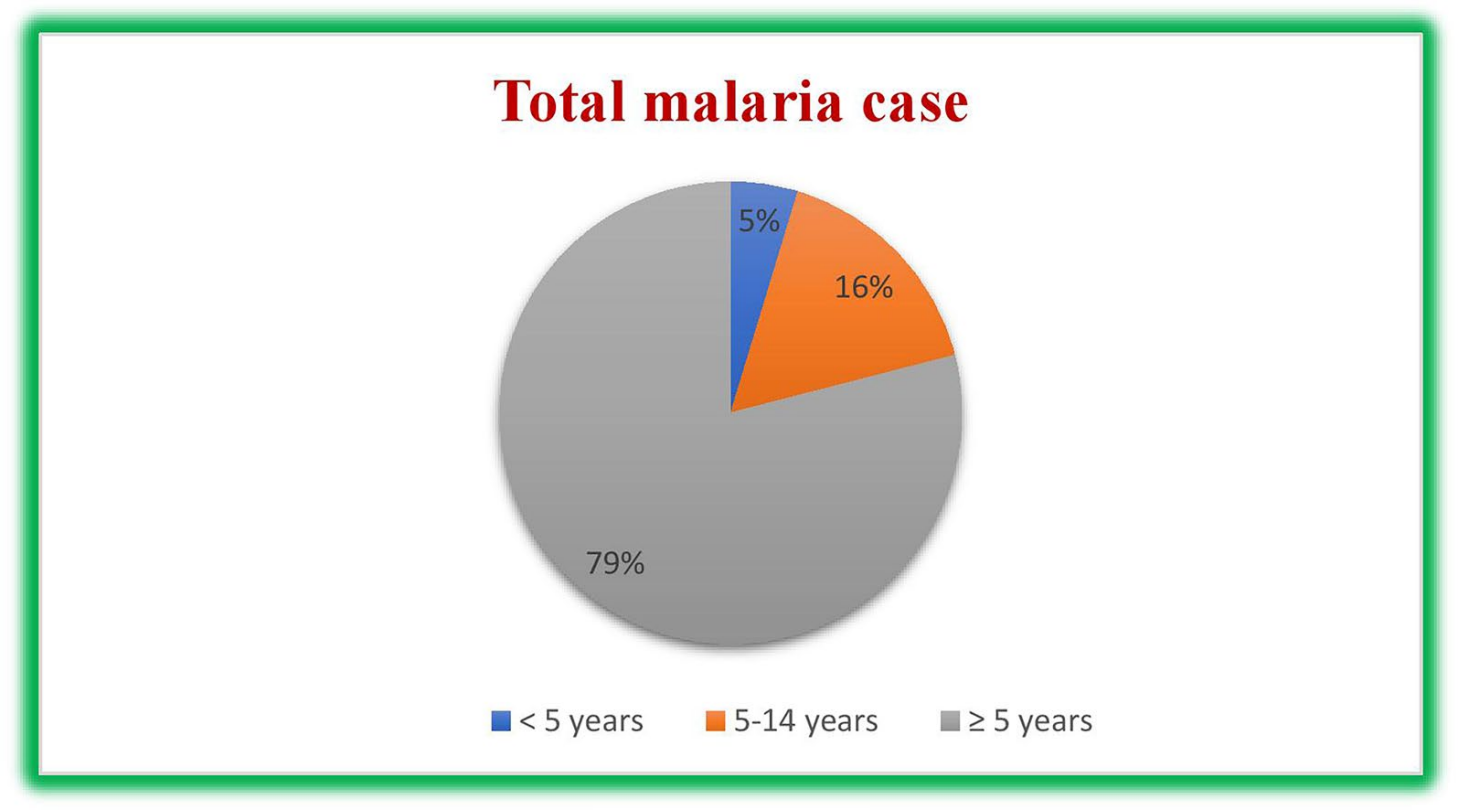
Malaria distribution by age in North Shewa zone, Ethiopia, July 2018–June 2023

Among all confirmed cases, 79% were individuals aged 15 years or older, while 16% and 5% were children aged 5–14 and 5 years, respectively. Additionally, as mentioned earlier, one death occurred within the age range of 5 to 15 years (Fig. 4).

#### Analysis by pregnancy status

**Fig. 5:**
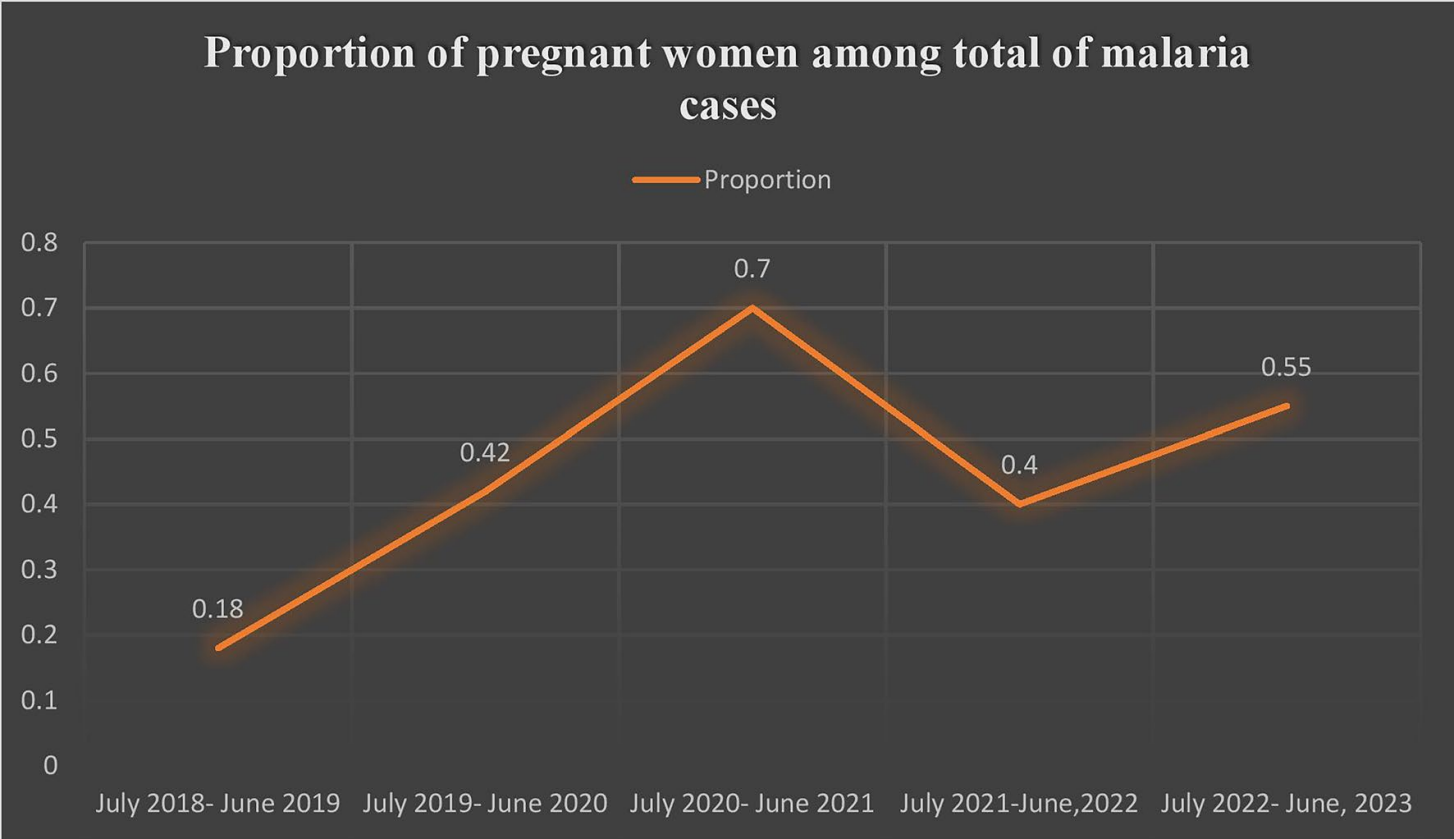
Proportion of pregnant women malaria cases in of North Shewa zone, July 2018–June 2023

Over the past 5 years, 224 cases (0.47%) out of the total malaria cases (47,889) were identified as pregnant women. From July 2018 to June 2021, there was a notable increase in the proportion of pregnant women diagnosed with malaria (Fig. 5).

### Malaria surveillance data analysis by place

#### Total malaria distributions by districts

**Fig. 6:**
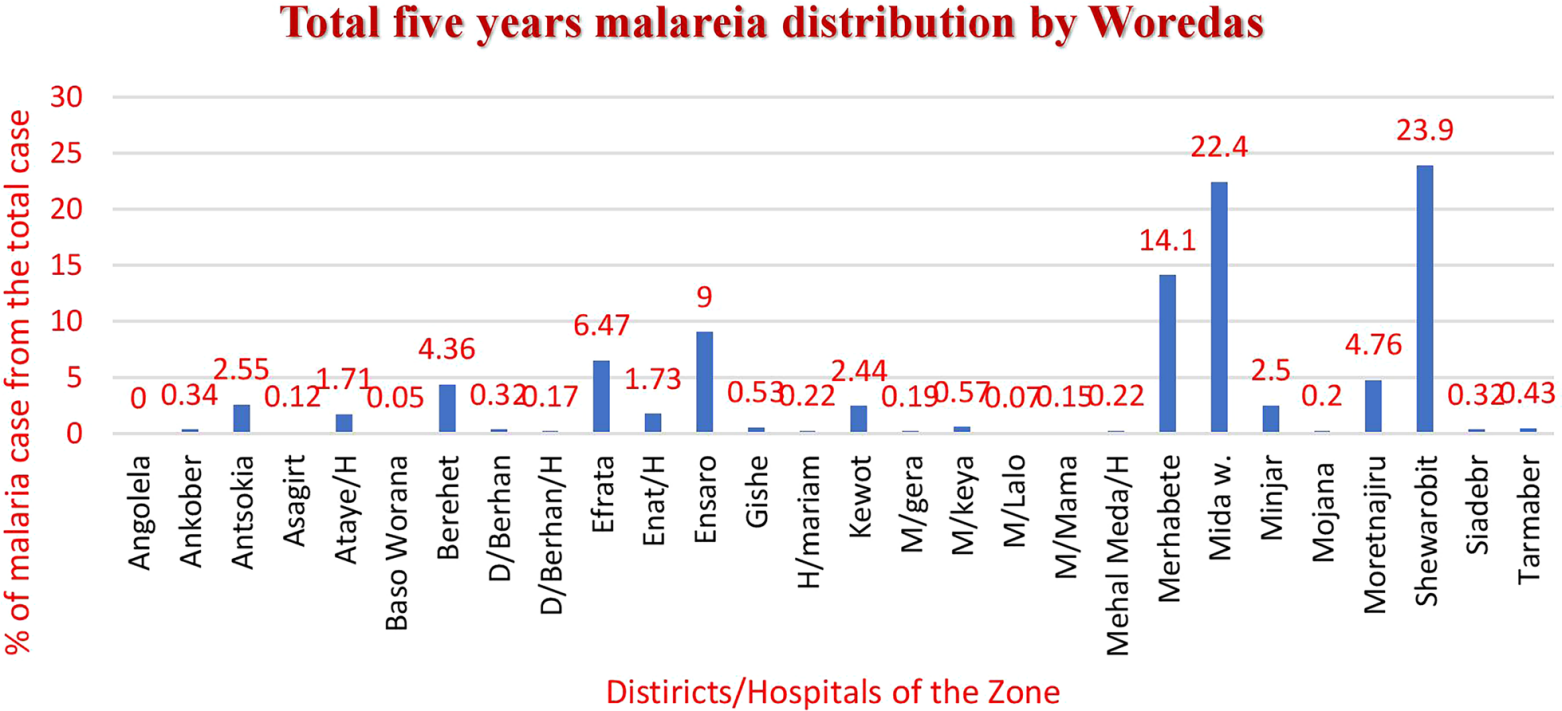
Total 5 years malaria distribution by place (districts) of North Shewa zone, July2018–June 2023

The analysis revealed malaria presence in all of the Zone’s woredas except Angolela. Over the past 5 years, three districts accounted for 60% of malaria cases in the zone: Shewarobit with 11,467 cases (23.9%), Mida-Woremo with 10,720 cases (22.4%), and Merhabete with 6,764 cases (14.1%). Conversely, the lowest number of malaria cases were reported from Basona Werna with 24 cases (0.05%), Menz Lalo with 37 cases (0.07%), and Asagrt with 56 cases (0.12%) (Fig. 6). Data from Debre Berhan town, Debre Berhan hospital, and Ataye hospital were unavailable for the Ethiopian fiscal year 2015 (July 2022 to June 2023) due to changes in reporting sites and a conflict situation at Ataye hospital.

#### Distribution of *Plasmodium* species by districts

**Fig. 7:**
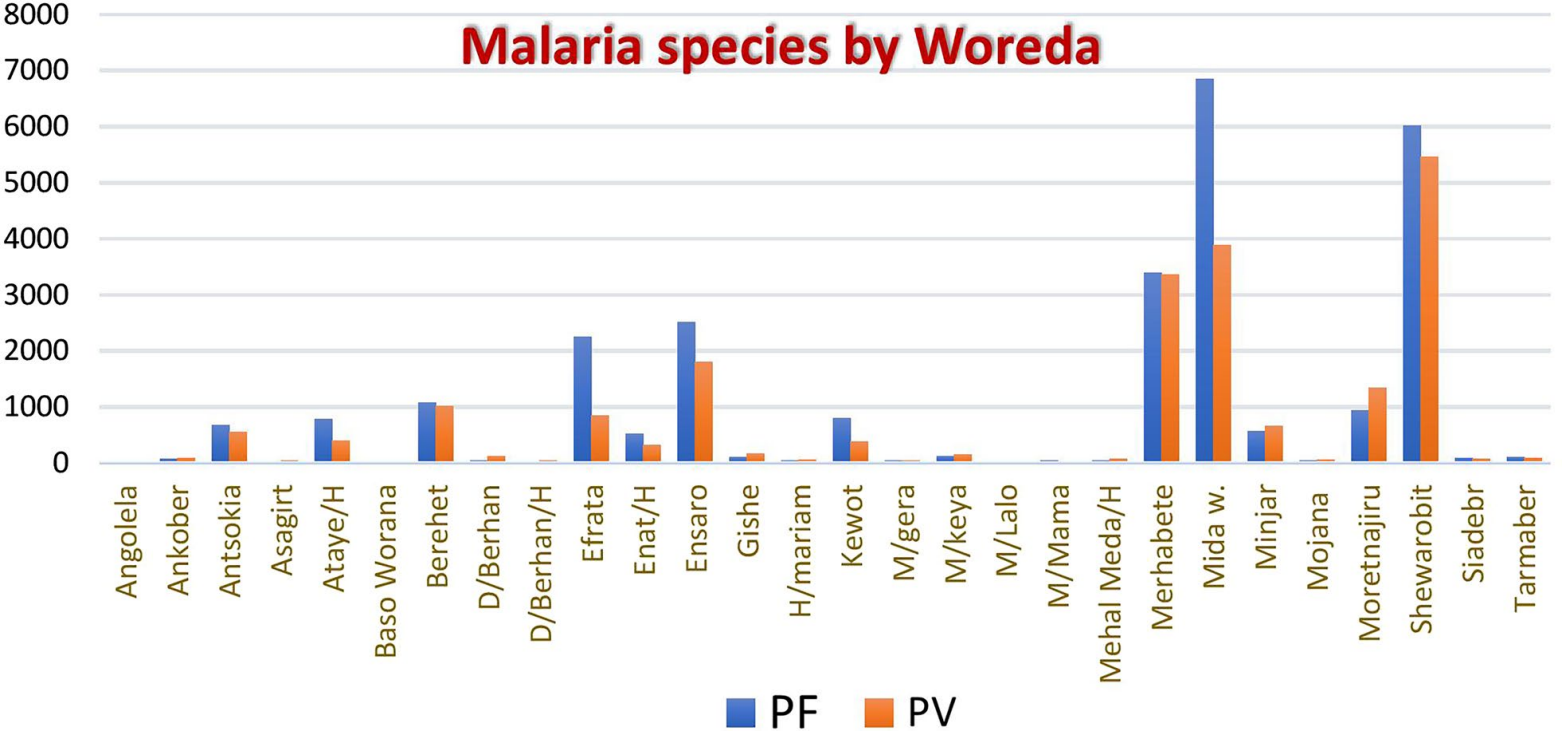
Malaria plasmodium species distribution by Woreda of the North Shewa zone, 2023

Regarding the distribution of parasite species in relation to woreda of the zone, Mida Woremo, Ensaro and Efratana Gidm woredas were more affected by *P. falciparum* than *P. vivax*. On the other hand, *P. falciparum* and *P. vivax* were evenly distributed in other woredas of the zone (Fig. 7).

## Discussion

Malaria is one of the major serious public health and life-threatening parasitic disease caused by Plasmodium species [35]. The peak malaria transmission season in the country is from September to December, following the main rainy season from June/July to early September [19, 20]. The result of this surveillance data analysis is in line with results from Kombolch town, Bahir Dar city, Adama, Mojo, Dembia, Northwest Tigray, Ataye, Jimma, and Harari [8, 9, 20–26]. The similarity could be explained by, these areas have similar raining season and following this the environment is comfortable for *Anopheles* mosquito breeding [10, 27].

This surveillance analysis result showed that, from the suspected fever cases 11.03% were malaria cases. Which is comparable with surveillance analysis result of East Shewa zone in Oromia region, 13.4% [28]. It is much lower than study in Guba district in Benshangul Gumuz, (51.04%) [29], Dembia (21.8%) [9], Sibu Sire (20.1%) [30], Harari (46.9%) [25], Abeshge (33.8%) [31] and Bale Zone (17.6%) [32]. This difference could be explained by most of the above are known malaria endemic areas and need further investigation climatic factors and the status of malaria interventions in the region [33] might have greatly contributed. Additionally, this can be explained by the fact that most of the aforementioned studies were conducted in single districts, such as Guba district in Benishangul Gumiz, which is known for its high prevalence of malaria. In contrast, the study site for the current research encompasses a broader area that includes all three agro-ecological zones. However, the results of this study were higher than the confirmed malaria cases reported in other similar retrospective studies conducted in Bahir Dar city (5.0%), Kombolcha town (7.52%), and Mojo town (4.2%) in Ethiopia [20, 26, 34]. The difference may be all Kombolcha, Bahir Dar and Mojo are urban and most of the population living in the area may have a good awareness on preventive practice of malaria than our study area population, most of them are rural.

*Plasmodium falciparum* is responsible for 60–70% of malaria cases in Ethiopia, with *P. vivax* causing the remainder [10]. A 5 years surveillance analysis in East Shewa zone showed that, from a total of confirmed malaria cases, 61% *P. falciparum* and 39% were *P. vivax* [28]. From confirmed malaria cases in Central, North and West Gondar zones; *P. falciparum* species were 73.4% and *P. vivax* were 26.6% [36]. In this surveillance analysis, *P. falciparum* were lower than those reported, that is 56.4% *P. falciparum* and 43.6% *P. vivax*. Whereas study in Mojo, Ethiopia, *P. vivax* was the predominant species accounting for 76.2% [26].

As the surveillance result showed in North Shewa zone within 5 years period > 15 years of age were more affected by malaria. Out of total confirmed and clinical malaria cases obtaining outpatient and inpatient service, 79% were age of 15 years and above, and the remaining 16 and 5% were children of age 5–14 and < 5 years, respectively. This is in line with finding from Gondar which showed that > 15 years of age accounts 71.89% followed by 5–14 years of age [36]. Another study in Adama also showed that 60% of all malaria positive cases were individuals with ≥ 15 years of age, followed by 5–14 age groups (28%), while children below the age of 5 were least affected [21]. This similarity may be explained by, adults are actively engaged in an agricultural activity that might expose them to an *Anopheles* mosquito bite. In addition, they travel far for schooling which also exposes them to infection [37].

## Conclusion and recommendation

According to 5 year PHEM data, the average annual malaria incidence rate in the Zone stood at 4.4 per 1000 population, indicating a high burden that makes achieving a malaria-free nation by 2030 challenging. *Plasmodium falciparum* malaria emerged as the predominant malaria parasite species in the zone. Malaria transmission typically begins in September and extends through December, and disproportionately affecting individuals aged 15 and above. In light of these findings, it is imperative to intensify malaria prevention and control efforts to reduce the malaria burden. Public Health Emergency Management officials at the Zone, Districts, and health facility levels should prioritize strengthening malaria data documentation and handling across all districts within the zone. This data should be rigorously analysed to promptly identify and respond to outbreaks, particularly during peak transmission seasons.

## Limitation of the study

The study uses the secondary data/surveillance data which lacks some important variables, like gender and other sociodemographic variables.

## Data Availability

All the data are available from the corresponding author upon reasonable request. All relevant data are within the manuscript.

## Abbreviations

PHEM: Public Health Emergency Management
RDT: Rapid Diagnostic Test
